# Ventricular Function and Tissue Characterization By Cardiac MRI in Children Following Hospitalization for Multisystem Inflammatory Syndrome in Children (MIS-C): A Prospective Study

**DOI:** 10.21203/rs.3.rs-1254952/v1

**Published:** 2022-01-24

**Authors:** Michael P Dilorenzo, Kanwal M Farooqi, Amee M Shah, Alexandra Channing, Jamie K Harrington, Thomas J Connors, Karen Martirosyan, Usha S Krishnan, Anne Ferris, Rachel J. Weller, Donna L Farber, Joshua D. Milner, Mark Gorelik, Erika B Rosenzweig, Brett R Anderson

**Affiliations:** CUIMC: Columbia University Irving Medical Center; CUIMC: Columbia University Irving Medical Center; CUIMC: Columbia University Irving Medical Center; CUIMC: Columbia University Irving Medical Center; CUIMC: Columbia University Irving Medical Center; CUIMC: Columbia University Irving Medical Center; CUIMC: Columbia University Irving Medical Center; CUIMC: Columbia University Irving Medical Center; CUIMC: columbia University Irving Medical Center; CUIMC: Columbia University Irving Medical Center; CUIMC: Columbia University Irving Medical Center; CUIMC: Columbia University Irving Medical Center; CUIMC: Columbia University Irving Medical Center; cuimc:Columbia University Irving Medical Center; CUIMC: Columbia University Irving Medical Center

**Keywords:** MIS-C, COVID-19, MOLLI, T1 mapping, Cardiac Magnetic Resonance Imaging, T2 mapping

## Abstract

**Background:**

Multisystem Inflammatory Syndrome in Children (MIS-C) is a severe life-threatening manifestation of SARS-CoV-2 infection. Acute cardiac dysfunction and resultant cardiogenic shock are common in children with MIS-C. While most children recover rapidly from acute illness, the long-term impact on the myocardium and cardiac function is unknown.

**Methods:**

In this prospective study, cardiac MRI (CMR) was performed on patients <21 years of age with a history of MIS-C, 6–9 months following hospitalization. Per institutional protocol, patients with any history of LVEF<50%, persistent cardiorespiratory symptoms, or ECG abnormalities underwent clinical CMR. Research CMRs were offered to all others >10 years old. Native T1 and T2 mapping values were compared with 20 children with normal CMR examinations.

**Results:**

We performed CMRs on 13 subjects at a median age of 13.6 years (interquartile range [IQR] 11.9–16.0) and a median time from hospitalization of 8.2 months (IQR 6.8–9.6). Twelve subjects displayed normal ventricular function with a median left ventricle ejection fraction (LVEF) of 57.2% (IQR 56.1–58.4) and median right ventricular (RV) EF of 53.1% (IQR 52.0–55.7). One subject had low normal EF (52%). There was normal T2 and native T1 as compared to normal controls. There was qualitatively no evidence of edema by T2 weighted imaging. One subject had late gadolinium enhancement (LGE) at the inferior insertion point and mid-ventricular inferolateral region, with normal EF, no evidence of edema or perfusion defects, and normal T1 and T2 times. When stratifying by a history of abnormal LVEF (LVEF <55%) on echocardiography, there was no difference in or parametric mapping values, though LVEF and LVEDV approached significance (p=0.06 and 0.05, respectively).

**Conclusions:**

Although many children with MIS-C present acutely with cardiac dysfunction, myocardial recovery is overall excellent with minimal to no evidence of residual cardiac dysfunction or myocardial involvement. LVEF by CMR at 6–9 months among children with history of echocardiographic LV dysfunction is slightly lower, though does not meet statistical significance and is still within normal range. The long-term functional implications of this finding and the cardiac implications of MIS-C more broadly are unclear and warrant further study.

## Background

While children were relatively spared from severe acute disease secondary to the initial strains of severe acute respiratory syndrome coronavirus-2 (SARS-CoV-2) infection, some developed a severe hyperinflammatory process associated with SARS-CoV2–2 infection, termed multisystem inflammatory syndrome in children (MIS-C).(1–4) This syndrome was characterized by high fevers, elevated inflammatory markers and in some, respiratory failure, significant cardiovascular compromise, including vasodilatory shock, severe valve regurgitation, ventricular dysfunction, and coronary artery dilatation.(3) Fortunately, most children recover rapidly from their acute presentation, with near-resolution of cardiac dysfunction and coronary dilatation by echocardiography by 4–9 months.(3)

While mid-term clinical and echocardiographic reports to date have been encouraging, echocardiography cannot characterize the myocardium for the presence of fibrosis or edema that may be associated with long-term cardiac dysfunction or poor outcomes. Considering the similar degrees of systemic inflammation and myocardial disease noted in other viral-induced myocarditis during initial hospitalization, evaluating for the presence of significant long-term myocardial involvement is necessary. (5, 6) Despite significant improvement in symptomatology and echocardiographic markers in patients with MIS-C, understanding myocardial involvement and persistent myocardial inflammation may have implications towards exercise restrictions, returning to play, and long-term well-being. Current joint AHA/ACC recommendations for competitive athletics recommend restricting sports participation until inflammation has subsided or at least 3–6 months following the diagnosis of myocarditis(5), and the American Academy of Pediatrics and others have used this to design guidelines for patients following MIS-C diagnosis. (6) There remains limited data on persistence of cardiac inflammation after MIS-C, and expert consensus to date has relied heavily on extrapolation from related conditions. A more nuanced understanding of long-term cardiac inflammation post MIS-C hospital discharge may aid in counseling patients and families on return to sports and long-term implications.

Cardiac magnetic resonance imaging (CMR) is the gold standard non-invasive tool for tissue characterization, and is now mainstay for diagnosing the presence of edema and fibrosis in the diagnosis of viral induced myocarditis.(7, 8) More recently, parametric mapping, including T1 and T2 mapping, has been employed as a method to identify and quantify diffuse fibrosis and edema.(9–11) T1 mapping has been shown to correlate with collagen volume fraction and extracellular matrix expansion as measured by histologic samples.(12–14) Clinically, this has been correlated with severity of diastolic dysfunction among adult patients with heart failure with preserved ejection fraction, as well as all-cause mortality, and parametric mapping has been incorporated into updated guidelines in the diagnosis of myocarditis.(15, 16)

To date, CMR publications in children recovering from MIS-C are predominantly within the initial 3 months following hospitalization, and larger studies on mid-term outcomes have not incorporated quantitative markers of fibrosis or edema. (17–22) We performed a prospective CMR study of pediatric patients hospitalized with MIS-C 6–9 months following hospitalization versus healthy controls to assess for residual myocardial disease.

## Methods

### Subject acquisition and consent

This study was approved by the Institutional Review Board at Columbia University Irving Medical Center. Children and adolescents <21 years of age with a history of hospitalization for MIS-C and no known prior cardiac disease were recruited for participation in this study. All children with suspected MIS-C are evaluated by the Columbia University Interdisciplinary MIS-C Team and offered longitudinal follow-up by the Columbia University Interdisciplinary MIS-C Follow-up Program. Diagnosis and management of MIS-C at our institution follows our institutional protocol and has been previously described.(1, 3) All suspected cases of MIS-C were evaluated and adjudicated to confirm accurate diagnosis.

By institutional protocol, all children with history of ventricular dysfunction (LVEF <50%), residual cardiac symptoms, including chest pain, palpitations, or exercise intolerance, persistently elevated inflammatory markers, or ECG changes/persistent dysrhythmia were referred for CMR 6–9 months following hospitalization for MIS-C. Families of all other children 10 years of older in the Interdisciplinary MIS-C Follow-up Clinic were approached for research CMR. This age cutoff was chosen to maximize the likelihood of a successful completion of the CMR exam without the need for sedation. Informed consent and assent were obtained for all subjects.

### CMR image acquisition

All CMR studies were performed on a 1.5T GE Explorer CMR scanner (GE healthcare systems, Chicago, IL, USA). The CMR protocol included conventional balanced steady state free procession (bSSFP) cine imaging in long and short axis planes using end-expiratory breath holding or apnea based on anesthesia requirement. Cine imaging slice thickness was modified to obtain 10–12 images per short axis stack. Pre-contrast T2 mapping was performed in basal, mid, and apical short axis slices using a breath-held or apnea motion corrected turbo spin echo sequence with a T2 preparatory pulse and bSSFP readout with the following typical parameters: echo times: 11 ms, 41 ms, 71 ms, 100 ms; slice thickness, 8mm; bandwidth 780Hz/pixel; pixel size, 2.2 × 2.2 mm; FOV (mm) 350; phase FOV (%), 70%; temporal resolution, 127 ms; phase resolution, 160 × 160; acceleration factor of 2. and T2-weighted turbo spin echo (TSE) in basal, mid, and apical short axis slices was acquired. Native T1 mapping was obtained using electrocardiogram triggered modified Look-Locker inversion recovery (MOLLI) sequences in basal, mid, and apical short axis slices during diastole using a 5(3)3 sequence. Typical sequence parameters were: slice thickness, 8mm; flip angle, 35°; echo time, 1.6 ms, repetition time, 3.8ms, bandwidth 1250Hz/pixel; pixel size, 2.2 × 2.2 mm; FOV (mm) 350; phase FOV (%), 70%; temporal resolution, 261 ms; phase resolution, 160 × 160; acceleration factor of 2. First pass perfusion imaging was performed immediately following the administration of 0.2mmol/kg of Gadobutrol. Phase sensitive inversion recovery imaging was performed in long and short axis planes to assess for late gadolinium enhancement (LGE). All static sequences except for first pass perfusion, were performed using breath holding or apnea (based on anesthesia needs) at end-diastole.

### CMR imaging post-processing

Image post-processing was performed using Circle CVI42 (Circle Cardiovascular Imaging Inc, Alberta, CA). Left and right ventricular (LV and RV, respectively) end-diastole and end-systole endocardial and epicardial borders were traced to determine end-diastolic and end-systolic volumes (EDV and ESV respectively and myocardial mass. Ejections fraction (EF) were calculated by the equation ((EDV-ESV)/EDV)*100 and reported as percentages. Indexed EDV, ESV, and masses were calculated by dividing by body surface area (BSA). Volume and LV mass Z-scores were generated using published data. Z-scores between 2.0 and 3.5 were considered mildly enlarged, between 3.5 and 5.0 were considered moderately enlarged, and >5.0 were considered severely enlarged.(23)

**Figure 1:**
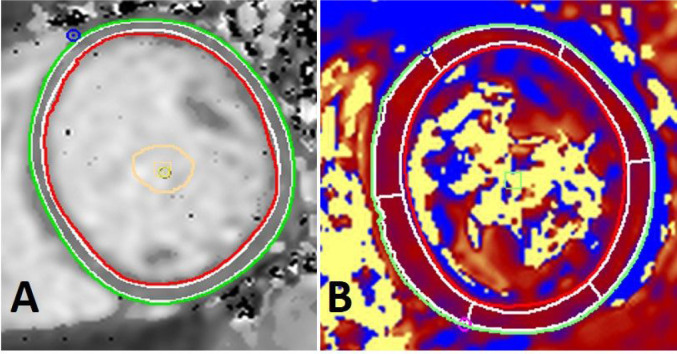
Native T1 and T2 tracings. Representative image of mid-ventricular native T1 and T2 map. Endocardial (red) and epicardial borders (green) are traced to avoid blood pool and pericardium, and 10% offset (white) to ensure only myocardium is contoured. Tracings are from a subject with normal T1 and T2 times.

T2 and T1 mapping were analyzed in the respective CVI42 modules. Endocardial and epicardial borders were traced with 10% offset to ensure that only myocardium was included in tracings (Figure 1). T2 and native T1 values (msec) are reported as the mean value for the given basal, mid-ventricular, or apical slice.

T2 TSE, first pass perfusion, and inversion recovery imaging were reviewed to determine the presence of edema, perfusion defects, and LGE, respectively. All imaging analyses were performed by a single pediatric cardiologist with extensive training and experience in pediatric CMR (MPD).

### Controls

Controls for native T1 and T2 mapping were retrospectively identified by review of the CMR database for patients with structurally normal hearts undergoing CMR to evaluate coronary anatomy with normal coronary origins, ECG abnormalities, or isolated PVCs/PACs to rule out arrhythmogenic RV dysplasia with normal biventricular size and systolic function and no evidence of LGE. CMR imaging performed solely to assess normal scanner T2 and native T1 times per Society of Cardiovascular Magnetic Resonance (SCMR) recommendation with qualitatively normal biventricular size and function were also included as controls.

### Statistical analysis

Descriptive statistics were presented as frequency counts and percentages for categorical variables and mean ± standard deviation (SD) or median (interquartile range; IQR) for continuous variables, as appropriate. Based on the distribution of the data, Student’s t-test or Wilcoxon rank sum was used to compare T1 and T2 values between subjects and controls. Spearman rho or Pearson correlation coefficient was used to assess associations between T1, T2, and LVEF as compared to lowest LVEF by echocardiography during hospitalization. The two-sided statistical significance level was set at 0.05. All data analyses were performed using STATA 14.1 (StataCorp, College Station, TX).

## Results

### Baseline data

**Figure 2:**
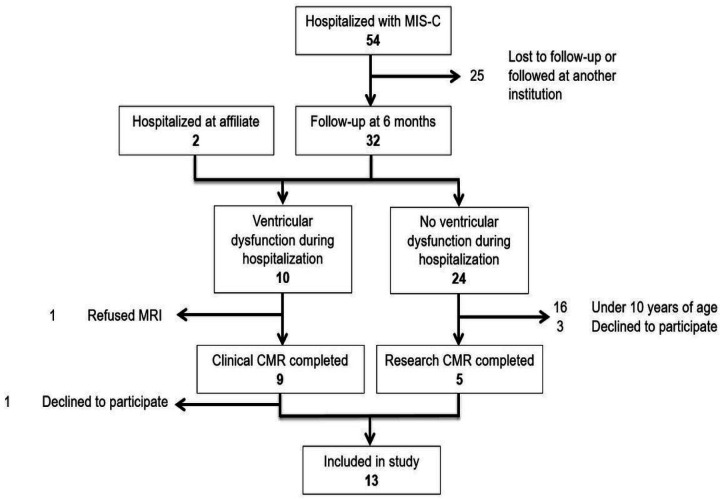
Patient flow plot. 54 patients were hospitalized at Morgan Stanley Children’s Hospital with MIS-C, with 12 ultimately undergoing CMR and 11 consenting for inclusion. Two additional patients hospitalized at an affiliate institution underwent CMR and were included in the analysis.

In total, 54 patients were admitted to Morgan Stanley Children’s Hospital with MIS-C between April 2020 and February 2021 and were eligible for inclusion. Thirty-two were followed for at least six months. Eight of these had cardiac dysfunction during initial hospitalization and, therefore, met clinical criteria for CMR. Of these, seven underwent CMR at our institution. Of these seven, one family declined to participate in this research. Two additional patients, hospitalized at an affiliated institution, were referred to our center for CMR. Of the eight children 10 years or older with normal cardiac function on admission followed for more than six months at our clinic, five agreed to participate (Figure 2).

In total, 13 subjects were included in this analysis (eight clinical and five research CMR) and underwent CMR. Two subjects (15%) required anesthesia with endotracheal intubation for CMR. Of the subjects who underwent CMR, 9 (69%) were male. The median age at hospitalization was 13.1 years (IQR 11.3–15.0), with a median length of hospital stay of 5 days (IQR 4–6). Nine (69%) subjects required ICU admission. Five (41 %) subjects required inotropic support and 5 (41 %) required respiratory support, including intubation in one subject. All subjects received IVIG, aspirin, and pulse steroids during the acute presentation with a steroid taper following discharge. LV dysfunction and coronary artery dilation or prominence was present in the majority (69% and 77%, respectively). Two subjects had residual ventricular dysfunction on discharge echocardiogram, which resolved by 3 months following discharge. Demographics and hospitalization data are described in Table 1.

### CMR data

CMRs were performed at a median of 8.2 months (IQR 6.8–9.6) months post hospital discharge and at a median age of 15.3 years (IQR 11.9, 16.4). Twelve subjects (93%) had follow-up echocardiograms around the time of CMR at a median of 6.4 (IQR 5.9–7.4) months following hospitalization. By echocardiography, eleven subjects had normal biventricular function, and one subject had mildly diminished biventricular function. All subjects were asymptomatic and had normal ventricular size with no evidence of significant mitral or tricuspid regurgitation.

On CMR, subjects had largely normal LV and RV ejection fraction (LVEF 57.2% (IQR 56.1–58.4); RVEF 53.1% (IQR 52.0–55.7)). Subjects overall had normal biventricular size and LV mass based on indexed ventricular size and mass and Z-score (Table 2). One subject had low normal LVEF (52%) with diminished apical wall motion, but otherwise normal ventricular size and mass, and no evidence of perfusion defects, LGE, or edema. Two subjects had mild LV dilation (Z-score +2.2 and +2.8) with overall normal LV and RVEF and no evidence of edema or fibrosis. T1 and T2 times in these subjects were normal.

There was no evidence of edema based on T2 TSE imaging and no perfusion defects noted on first pass perfusion. One subject—different from the aforementioned—had LGE at the inferior insertion point and mid-ventricular inferolateral region, with normal ventricular function, no evidence of edema or perfusion defects, and normal T1 and T2 times. This subject had a prior history of moderate LV dysfunction by echocardiography, with an LVEF of 44%. Characteristics of the two subjects with diminished function and LGE are presented in Table 3

**Figure 3:**
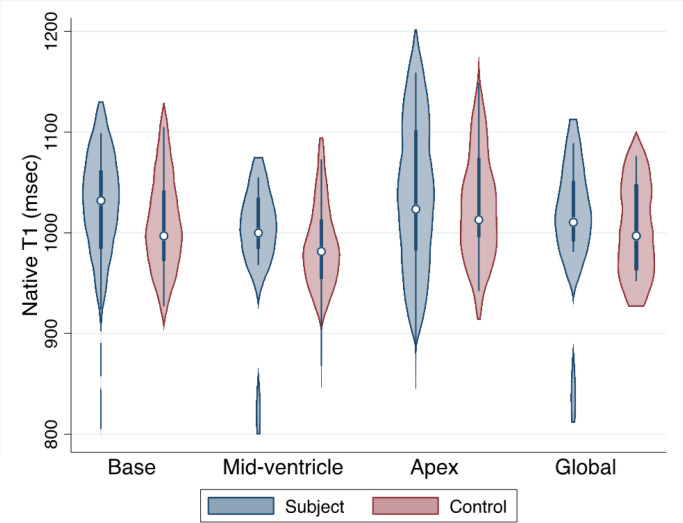
Native T1 values in subjects vs controls. Violin plot demonstrating native T1 values in subjects as compared to controls in basal (left) mid-ventricle (middle) and apical (right) short axis slices. Subjects (n=13) are in blue and controls (n=20) are in red.

**Figure 4:**
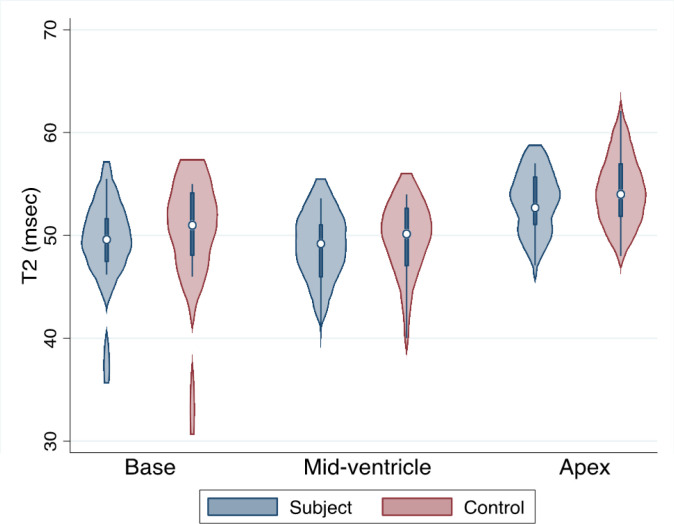
T2 values in subjects vs controls. Violin plot demonstrating T2 values in subjects as compared to controls in basal (left) mid-ventricle (middle) and apical (right) short axis slices. Subjects (n=13) are in blue and controls (n=15) are in red.

T2 and native T1 times are presented in Table 2. There was no difference in T2 or native T1 times as compared to controls (Table 2, Figure 3 and 4).

When stratifying subjects by ventricular function during initial admission, LVEF and LVEDV medians on CMR were marginally lower but not statistically different among those with and without a history of ventricular dysfunction (56% vs. 59%, p=0.06; 129mL vs. 199mL, p=0.05, respectively). There was no difference in indexed ventricular volumes, RVEF, or T1 and T2 mapping values (Table 4). Subjects with a history of low LVEF on admission echocardiogram tended to be younger (p=0.03). Among the 11 subjects with available LVEF on echocardiography during admission, there was no association between the lowest available EF and LVEF by CMR (Spearman rho 0.42, p=0.2).

## Discussion

The emergence of SARS-CoV-2 has resulted in significant morbidity and mortality across the globe. We are only just starting to ascertain the long-term impact that this new virus will have on human health. Acute cardiac dysfunction is a prominent and critical manifestation for children who develop MIS-C following infection with SARS-CoV-2. Whether acute cardiac dysfunction during MIS-C ultimately results in permanent cardiac dysfunction or persistent inflammation as seen in other viral induced myocarditis syndromes remains undefined. Determining the presence of cardiac inflammation and myocardial damage has significant implications towards counseling, as the presence of residual inflammation may necessitate restrictions from sports participation until such inflammation has resolved.Improving our understanding of the potential lifelong impact that MIS-C will have on children’s cardiac health is therefore essential to our ability to guide families and determine treatment plans.

We report on the mid-term CMR findings in children 6–9 months following hospitalization for MIS-C. Subjects overall have normal ventricular size and systolic function, with LV dysfunction present in only one subject. There is no evidence of edema and parametric mapping values were normal as compared to controls. LGE was present in only one subject with no evidence of perfusion defects and overall normal T1 times in this subject. Importantly, we show near-complete recovery in ejection fraction among subjects with a history of ventricular dysfunction.

Our group previously reported on six-month echocardiographic findings in this population and demonstrated excellent recovery in almost all patients.(3) While echocardiography is a valuable tool to assess global ventricular function, CMR remains the gold standard measure to assess myocardial tissue characterization.(9) Furthermore, LVEF by echocardiography requires numerous assumptions on LV geometry, and assessment of RV function is predominantly qualitative; CMR allows for a more granular calculation of EF.

Early CMR data among patients hospitalized with MIS-C has previously been reported, with variable degrees of edema and LGE, as well as diminished LV strain in those with LV dysfunction.(17, 20, 22) These findings are consistent with our prior data demonstrating significant functional impairment in many children with MIS-C.(1, 3) Our CMR cohort demonstrated a similar degree of dysfunction during hospitalization, with almost half of our subjects having had LV dysfunction by echocardiography.

Webster et al. previously reported on early term outcomes among children with a history of symptomatic COVID 1–3 months following presentation, including 6 subjects with MIS-C.(18) They demonstrated globally normal ventricular function and parametric mapping values by CMR among the 6 MIS-C patients, though prior cardiac dysfunction in their cohort was isolated to two subjects with history MIS-C. Similarly, Capone et al. demonstrated normal LV systolic function and no evidence of edema or fibrosis among 11 subjects 4–6 weeks following initial hospitalization for MIS-C.(19) Our findings are consistent with theirs, with a larger MIS-C sample size further from hospitalization. Our data further reinforces the narrative that recovery is overall excellent.

Beyond presenting prospective data further from hospitalization, our study is novel in that we incorporated the use of a parametric mapping for myocardial tissue characterization in a cohort exclusively comprised of a MIS-C population. Parametric mapping is quickly becoming a standard of care method in the evaluation of myocardial edema and diffuse fibrosis in patients, and has recently been incorporated as part of revised Lake Louise criteria in the evaluation of myocarditis.(7) Webster et al. evaluated native T1 and T2 values in pediatric patients with symptomatic COVID; however, they did not administer contrast in their cohort, and therefore were unable to assess for LGE. While Barris et al report on mid-term CMR findings in patients hospitalized with MIS-C, they did not perform parametric mapping; evaluation of edema and fibrosis was therefore limited to a qualitative analysis. Furthermore, while LGE can identify focal or replacement fibrosis, it is unable to identify diffuse fibrosis.(11, 18, 21)

There are several limitations to this study. Our sample size is limited, due to an *a priori* decision to only obtain clinical CMR to patients who had evidence of cardiac dysfunction during initial presentation and few subjects willing to consent to a research CMR with contrast administration. Therefore, we may have been underpowered to detect differences in functional and myocardial tissue characterization parameters. Furthermore, younger subjects were excluded from research CMR; as a result, we may not be able to extrapolate our findings to those hospitalized at a younger age. That said, our sample is biased to those with initial cardiac dysfunction, and therefore would be biased to those most at risk. Finally, control data were available for native T1 and T2 values as part of recommendations by SCMR to obtain local references;(11) as control data were based on subjects referred for clinical CMR, it is possible that myocardial tissue abnormalities were present. However, we ensured that all subjects used as controls had normal biventricular size and systolic function with no evidence of identifiable congenital heart disease or systemic diseases. Therefore, the likelihood of myocardial abnormalities is minimal. As availability of normal control myocardial T1 data were limited, we could not age match in this study, and therefore it is possible that lack of differences between controls and subjects was due to age dependent phenomenon. Furthermore, normal controls were not available for ECV due to the need for contrast administration. While comparisons to published reference values have been deemed acceptable, the availability of published data in children are limited to small single center cohorts.(11)

## Conclusions

Our study demonstrates overall excellent midterm cardiac outcomes among this cohort of children with MIS-C. Recovery of ventricular function was nearly universal with minimal evidence of residual myocardial disease in our cohort. Long-term, larger-scale studies are needed to ensure complete recovery in those with residual disease and to identify if any children remain at risk for long-term myocardial disease and dysfunction after MIS-C.

## Figures and Tables

**Table 1:** Baseline characteristics and demographics

	median (iqr) or frequency (%)	Range

Gender (male)	9 (67)	

Race	2 (15)	
White	5 (39)	
Black/African American	3 (23)	
Other	3 (23)	
Declined		

Ethnicity	6 (46)	
Hispanic/Latino	5 (39)	
Not Hispanic/Latino	2 (15)	
Declined		

Age at hospitalization	13.1 (11.3–15.0)	5.2–16.2

CMR indications	7 (54)	
Diminished LVEF	1 (8)	
Heart block	5 (38)	
Research		

Time from hospitalization to CMR (months)	8.2 (6.8–9.6)	

Length of hospitalization (days)	5 (4–6)	3–32

Symptoms (n=12)	12 (100)	
Fever	6 (50)	
Shortness of breath	2 (18)	
Cough	11 (92)	
Nausea/vomiting	10 (83)	
Diarrhea	8 (67)	
Rash	4 (33)	
Malaise	4 (33)	
Conjunctivitis	5 (42)	
Headache		

Intensive Care Unit stay	9 (69)	

Inotropic support	5 (41)	

Respiratory support	1 (8)	
Intubation	4 (33)	
CPAP/BiPAP or HFNC		

LV function (n=12)	4 (31)	
Normal	6 (46)	
Mildly diminished	2 (23)	
Moderately diminished		

Coronary dilation/prominence	10 (77)	

Mitral regurgitation	7 (54)	
Trivial	2 (15)	
Mild	1 (8)	
Mild to moderate	2 (15)	
Moderate	1 (8)	
Severe		

Tricuspid regurgitation	5 (38)	
Trivial	4 (31)	
Mild	2 (15)	
Mild to moderate	1 (8)	
Moderate	1 (8)	
Severe		

Data presented as median (IQR) or frequency (%) as appropriate. Symptoms are based on available report. Echo parameters are qualitative based on echo report. CMR, Cardiac Magnetic Resonance; LV, left ventricle; HFNC, high flow nasal cannula; CPAP, Continuous positive airway pressure; BiPAP, bilevel positive airway pressure

**Table 2:** CMR data

	Subjects	Controls	p-value
Age at CMR (years)	15.3 (11.9, 16.4)		
LVEF (%)	57 (56–58)		
RVEF (%)	53 (52–56)		
LVEDV (mL)	139 (128.1–191.3)		
Indexed LVEDV (mL/m2)	84.2 (76.5–97.7)		
LVEDV Z-score	−0.4(−1.0– 0.9)		
LV Mass (g)	64.7 (51.9–78)		
Indexed LV Mass (g/m2)	41.9 (36.7–43.5)		
RVEDV (mL)	147.4 (130.1–188.7)		
Indexed RVEDV (mL/m2)	91 (77.6–103.6)		
RVEDV Z-score	−1.0 (−2.0– 0.4)		
Native T1 (ms)			
Base	1032 (984–1062)	997 (972–1042)	0.61
Mid	1000 (984–1035)	981 (954–1014)	0.22
Apex	1024 (983–1102)	1013 (996–1074)	0.92
Global	1010 (991–1051)	997 (963–1048)	0.42
T2 mapping (ms)			
Base	49.6 (47.4–51.7)	51.0 (48.0–54.2)	0.53
Mid	49.2 (45.9–51.1)	50.2 (47.0–52.7)	0.63
Apex	52.7 (51.0–55.8)	54.0 (51.8–57)	0.32

Data presented as median (IQR). CMR, Cardiac magnetic resonance; LV, left ventricle; RV, right ventricle; EF, ejection fraction; EDV, end-diastolic volume; ECV, extracellular volume

**Table 3:** Characteristics of patients with left ventricular dysfunction and/or LGE on CMR

Subject	Age/gender at CMR	Time from admission to CMR (mo)	Admission LV function	Time to EF recovery	Length of hospitalization	ICU?	CMR LVEF	CMR RVEF	LGE location	Edema	ECV	Global native T1	Perfusion defects?
1	15.4 (M)	9.7	Moderately diminished	8 days	9 days	No	52%	53%	None	None	22%	1047	None
2	6.1 (M)	11.5	Moderately diminished	4 days	5 days	Yes	57%	50%	Inferior insertion, mid-ventricular inferolateral wall	None	23%	981	None

**Table 4:** CMR data among subjects with history of LV dysfunction vs normal LV function during hospitalization

Parameter	Low LVEF (n=7)	Normal LVEF (n=6)	P value
Age at CMR (years)	11.9 (6.1–15.3)	16.2 (13.1–16.7)	0.03
LVEF (%)	56 (55–58)	59 (57–61)	0.06
RVEF (%)	53 (52–54)	55 (51–59)	0.39
LVEDV (mL)	128.9 (81–153)	199.2 (139–212.8)	0.05
Indexed LVEDV (mL/m2)	84.2 (72.3–93.5)	90.8 (80.1–116.4)	0.39
LV mass (g)	61.0 (44.4–68.6)	76.2 (51.9–96.5)	0.20
Indexed LV mass (g/m2)	41.3 (36.7–42.4)	42.9 (31.4–45.2)	0.78
RVEDV (mL)	140.1 (86.3–149.4)	207.0 (130.1–235.7)	0.12
Indexed RVEDV (mL/m2)	88.2 (77.6–92.3)	99.8 (74.9–114.8)	0.32
Native T1 (ms)			
Base	1032 (975–1036)	1028.5 (993–1090)	0.63
Mid	988 (977–1019)	1020 (996–1043)	0.20
Global	1002 (990–1047)	1022 (993–1083)	0.52
T2 mapping (ms)			
Base	49.6 (46.2–53.7)	49.6 (48.4–51.6)	0.89
Mid	48.1 (45.6–52.5)	49.9 (48.1–51.1)	0.62
Apex	52.7 (51–55.7)	53.7 (51–56.6)	0.75

Data presented as median (IQR). CMR, Cardiac magnetic resonance; LV, left ventricle; RV, right ventricle; EF, ejection fraction; EDV, end-diastolic volume; ECV, extracellular volume

## List Of Abbreviations

BSA: Body Surface Area
bSSFP: balanced steady state free procession
CMR: Cardiac Magnetic Resonance
ECV: Extracellular Volume
EDV: End-diastolic Volume
EF: Ejection Fraction
ESV: End-systolic Volume
IQR: Interquartile range
LGE: Late Gadolinium Enhancement
LV: Left Ventricle
MIS-C: Multisystemic inflammatory syndrome in children
MOLLI: Modified Look-Locker Inversion
RV: Right Ventricle
SARS-CoV-2: severe acute respiratory syndrome coronavirus-2
SCMR: Society of Cardiovascular Magnetic Resonance
TSE: Turbo Spin Echo
